# UV Spectrophotometric Determination and Validation of Hydroquinone in Liposome

**Published:** 2015

**Authors:** Rabea Khoshneviszadeh, Bibi Sedigheh Fazly Bazzaz, Mohammad Reza Housaindokht, Azadeh Ebrahim-Habibi, Omid Rajabi

**Affiliations:** a *Department of Biology, Science and Research Branch, Islamic Azad University, Tehran, Iran. *; b *Biotechnology Research Center, Drug and Food Control Department, School of Pharmacy, Mashhad University of Medical Sciences, Mashhad, Iran. *; c *Chemistry Department, Faculty of Sciences, Ferdowsi University of Mashhad, Mashhad, Iran.*; d *Endocrinology and Metabolism Research Center, Endocrinology and Metabolism Research Institute, Tehran University of Medical Sciences, Tehran, Iran. *; e *Biosensor Research Center, Endocrinology and Metabolism Molecular-Cellular Sciences Institute, Tehran University of Medical Sciences, Tehran, Iran. *; f *Drug and Food Control Department, School of Pharmacy, Mashhad University of Medical Sciences, Mashhad, Iran.*

**Keywords:** Hydroquinone, Liposome, UV spectroscopy, Validation

## Abstract

The method has been developed and validated for the determination of hydroquinone in liposomal formulation. The samples were dissolved in methanol and evaluated in 293 nm. The validation parameters such as linearity, accuracy, precision, specificity, limit of detection (LOD) and limit of quantitation (LOQ) were determined. The calibration curve was linear in 1-50 µg/mL range of hydroquinone analyte with a regression coefficient of 0.9998. This study showed that the liposomal hydroquinone composed of phospholipid (7.8 %), cholesterol (1.5 %), alpha ketopherol (0.17 %) and hydroquinone (0.5 %) did not absorb wavelength of 293 nm if it diluted 500 times by methanol. The concentration of hydroquinone reached 10 µg/mL after 500 times of dilution. Furthermore, various validation parameters as per ICH Q2B guideline were tested and found accordingly. The recovery percentages of liposomal hydroquinone were found 102 ± 0.8, 99 ± 0.2 and 98 ± 0.4 for 80%, 100% and 120% respectively. The relative standard deviation values of inter and intra-day precisions were <%2. LOD and LOQ were 0.24 and 0.72 µg/mL respectively.

## Introduction

**Figure 1:**
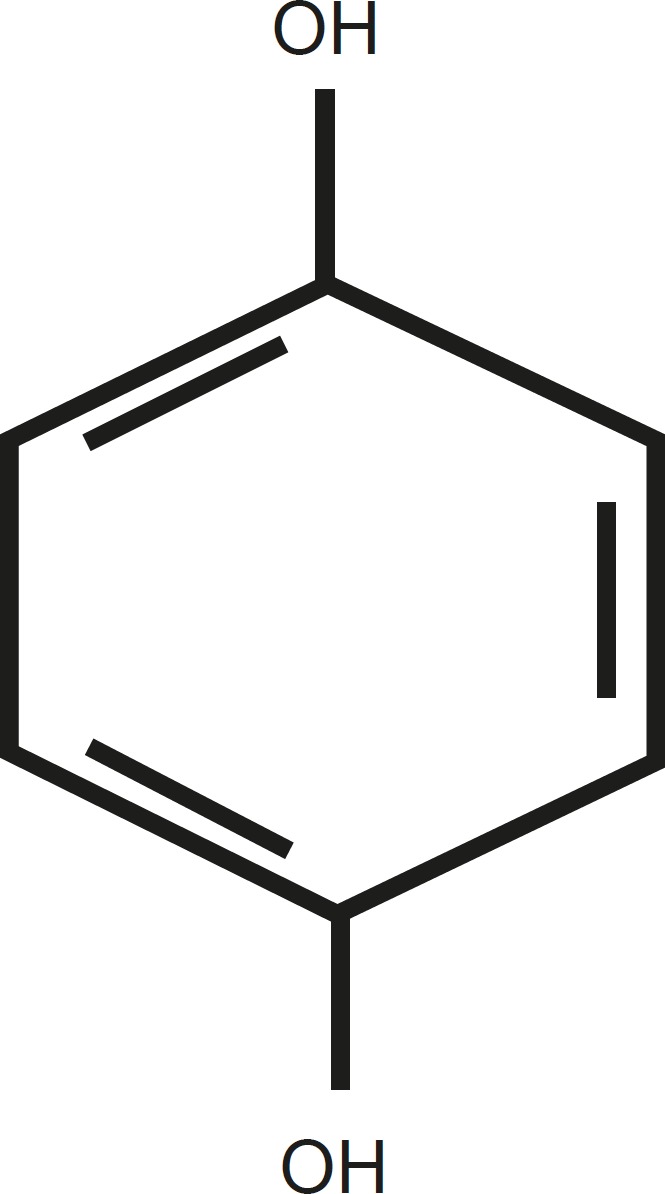
The structure of Hydroquinone

Hydroquinone (HQ) (1, 4-benzenediol; C_6_H_4_ (OH)_ 2_) is a white crystalline substance. It is highly soluble in water (70 g/L at 25 °C) and the log n-octanol/water partition coefficient is 0.59 (Figure 1).

Hyperpigmentation skin disorder is treated with HQ products. This substance inhibits tyrosinase enzyme which is responsible for the first reaction of melanin formation (1). Subsequently melanin decreases and skin becomes depigmented.

Topical drugs have to cross from stratum corneum layer of skin that acts as a barrier against skin permeation (2). For solving this problem, these drugs are formulated with carrier systems that are able to increase skin permeation (3). There are reports on different topical liposomes with effective drug or analyte delivery to the skin, such as Amphotricin B (4), Clarithromycin (5), Safranal (6), Octyl Methoxycinnamte (7), alpha tocopherol (8).

So far, the formulations of HQ cream, gel, lotion, and solution are provided and are developed by several techniques to determine HQ. The spectrophotometry, high-performance liquid chromatography (HPLC), thin layer chromatography (TLC), micellarelectrokinetic chromatography (MEKC), and capillary electrochromatography (CEC) techniques (9-19) determine HQ in various matrices such as air, waste photographic solutions, cream, and lotion. Fortunately, HQ could be determined by spectrophotometry technique that is fast, available and simple.

The objective of this study was to determine HQ in liposomal samples by spectrophotometry and to obtain validation parameters.

## Experimental

*Material and methods*

Phospholipid S 100 (Phosphatidylcholine (PC) from soybean Lecithin) was obtained from Lipoid Company. HQ, cholesterol, sodium metabisulfite and chloroform (analytical grade) were purchased from Merck. Alpha ketopherol acetate and methanol (analytical grade) were purchased from Applichem and Chemical Pars respectively.

*Instrumentation*

Buchi rotary evaporator (R-210), IKA T10 hemogenaizer, Cecil 9500 Double Beam Spectrophotometer and Shimadzu balance with 0.1 mg accuracy were used through the experiments.

*Preparation of HQ liposomes*

Liposome dispersion samples were prepared by a chloroform film method [20] with homogenization. Liposome formulations composed of S100(7.78 %), cholesterol (1.5 %), alpha ketopherol (0.17 %) and HQ (0.5 %) w/v were dissolved in 15 mL chloroform and 5 mL methanol. Thin film layer was formed by vacuum-desiccated the solution by Buchi rotary evaporator (R-210), then was flushed with nitrogen gas for 1 min. The thin film was re-suspended in solution of 0.01M phosphate buffer (pH 6) with sodium metabisufite (0.1%) slowly, and swelled by shaking hand, vortex and final liposome solution homogenized for 5 min by 2000 rpm speed of IKA T10 homogenizer.

*Preparation of HQ-free of liposome (placebo)*

 The preparation of HQ-free of liposome was the same preparation of HQ liposome, but HQ was not added to primary solution.

*Preparation of HQ standard solutions*

For construction of the calibration curve, accurately weighed HQ (500 mg) was transferred to a 100 mL volumetric flask and dissolved in distilled water. 2 mL from this solution transferred to 100 mL volumetric flask and complete the volume with methanol, stock solution was obtained. Stock solution diluted by methanol to obtain 1, 8, 10, 12, 20, 30, 40, 50 µg/mL concentrations (triplicate). The standard solutions were prepared daily.

For the precision studying, hydroquinone 5 mg/mL solution in water was prepared then it was diluted 500 times by methanol to obtain a final concentration of 10 µg/mL (triplicate).

*UV method*

Accurate volume of HQ liposome (equivalent to 5 mg of HQ) was transferred to a 50 mL volumetric flask and dissolved in methanol to obtain a concentration of 100 µg/mL. An aliquot of this solution was diluted in methanol to obtain a solution with final concentration of 10 µg/mL.

*Method validation*

For validation studying, the International Conference on Harmonization (21) and AOAC International Guidelines (22) for Validation of Analytical methods were used. 

*Linearity*

The calibration curve was obtained at eight concentration levels of HQ solutions (1-50 µg/mL). By the least square regression method the linearity was evaluated with triplicate determinations at each concentration level.

*Precision*

The degree of aggregate among test results was controlled when a method was applied repeatedly. Variation in intra-day and between days (inter-day) was analyzed. The intra-day and inter-day precision was determined by analyzing same concentration of HQ (10 μg/mL).

*Accuracy*

An accurate volume of HQ liposome (equivalent to 5 mg of HQ) was transferred to a 50 mL volumetric flask and dissolved in methanol (100 µg/mL). Aliquots of this solution and 3, 5 and 7 mL of a HQ stock solution (100 µg/mL) were transferred into 100 mL volumetric flasks and then methanol was added to make up the volume to give final concentrations of 8, 10 and12 µg/mL. All solutions were prepared in triplicate and analyzed.

*Specificity*

The specificity was evaluated by analyzing HQ-free of liposome, where in the sample matrix was analyzed without the analyte. The system result was examined for the presence of interferences or overlaps with the HQ result. HQ-free of liposome solution (placebo) were diluted 10, 100 and 500 times by methanol and their spectrums was obtained.

*Limit of detection (LOD) and limit of quantitation (LOQ)*

The limit of detection (LOD) of HQ was evaluated from the slope (S) of calibration curve and the standard deviation of the blank (δ) using equation as (23)

LOD =3.3 δ/S

The minimum quantity of drug that can be quantified by the instrument is defined LOQ. The LOQ were evaluated from the slope(S) of calibration curve and the standard deviation of the blank (δ) using equation as:

LOQ = 10 δ/S

## Results and Discussion

The UV is a rapid and easy method for determination of HQ in liposome. The range of calibration curve was constructed in 1-50 µg/mL in293 nm. The method was validated according to ICH Q2B Guidelines for validation of analytical procedures in order to determine the linearity, LOD, LOQ, precision and accuracy for the HQ. 

*Linearity and range*

**Figure 2:**
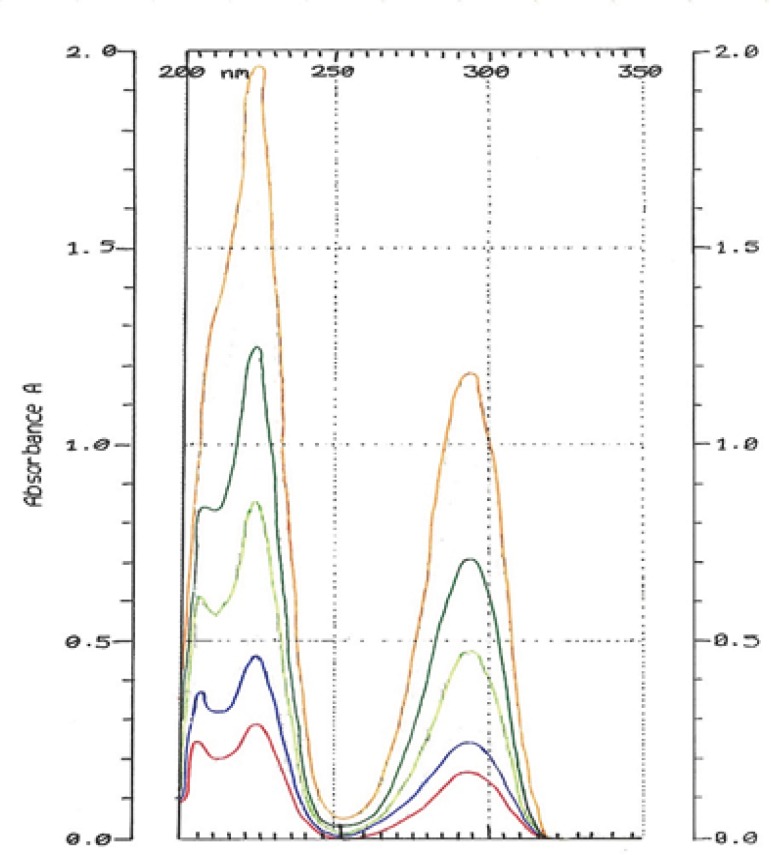
Overlay of hydroquinone standard solutions in different concentrations

**Figure 3:**
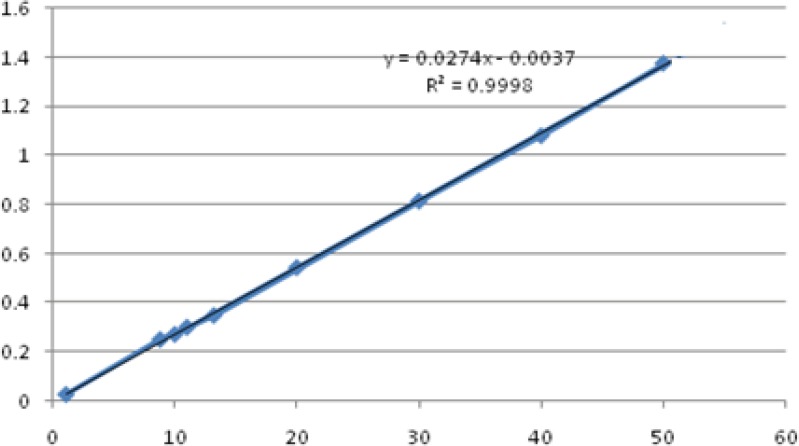
Calibration curve of hydroquinone standard solutions in 1, 8, 10, 12, 20, 30, 40, 50 µg/mL concentrations

The spectrums of HQ standard solutions were obtained (Figure 2) and the linearity was determined by plotting standard calibration curve for the concentration range 1-50 μg/mL as shown in Figure 3.

The Correlation Coefficient was *r*^2 ^= 0.9998 that showed excellent linearity.

*Specificity*

**Figure 4:**
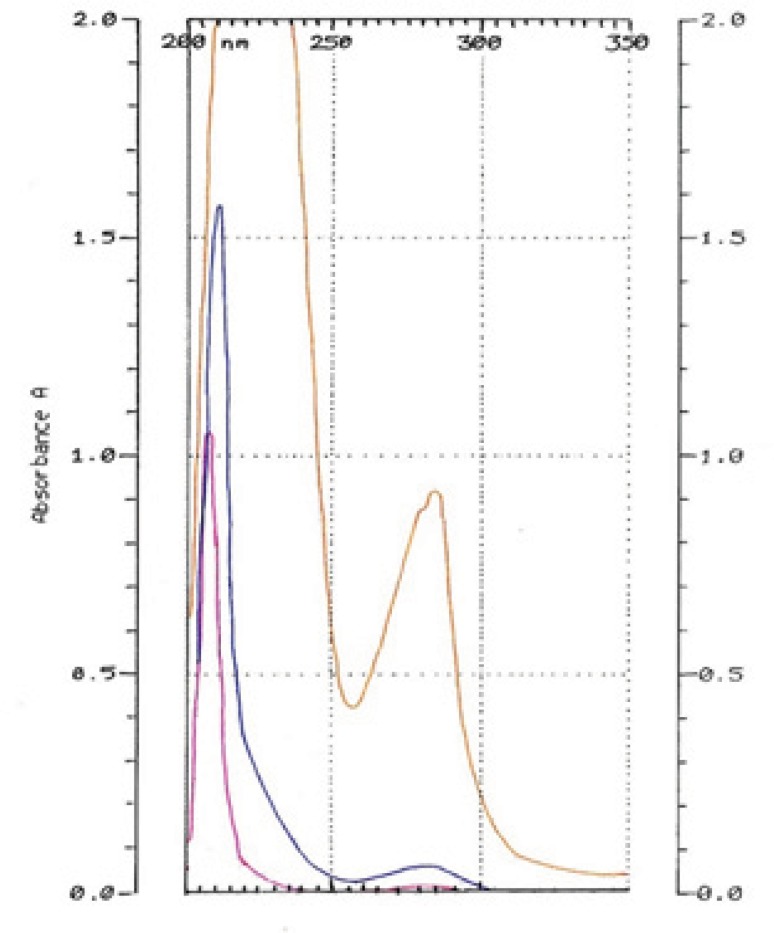
Hydroquinone-free Liposome Spectrums in 10 (orange), 100 (blue) and 500 (pink) time dilutions.

**Figure 5:**
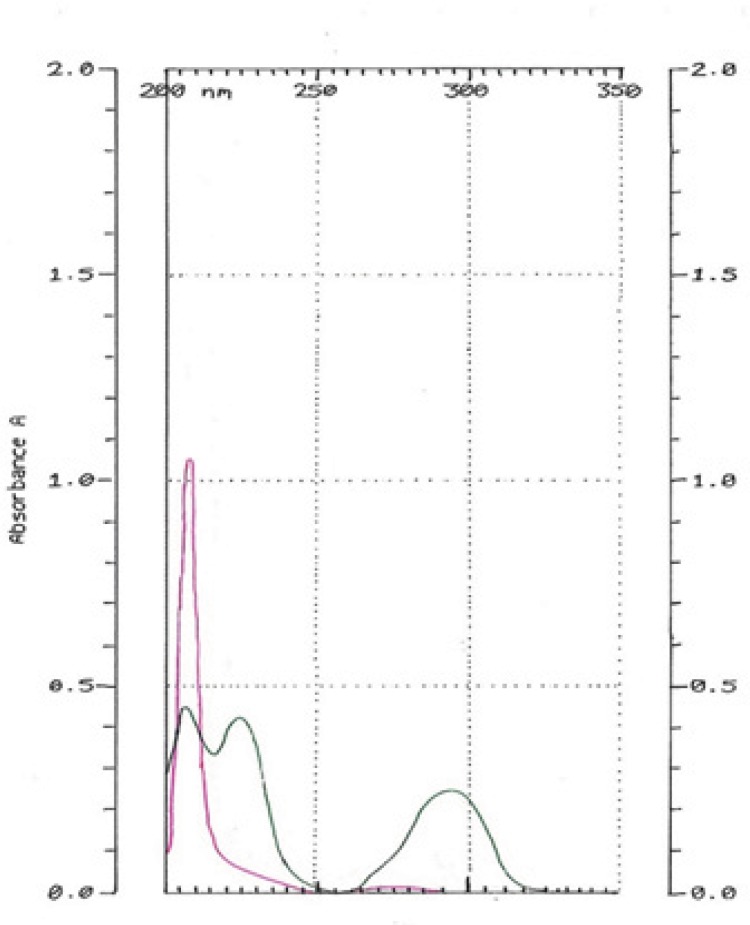
Hydroquinone-free liposome solution diluted 500 times (pink). HQ standard solution in 10 µg/mL concentration (green).

**Figure 6:**
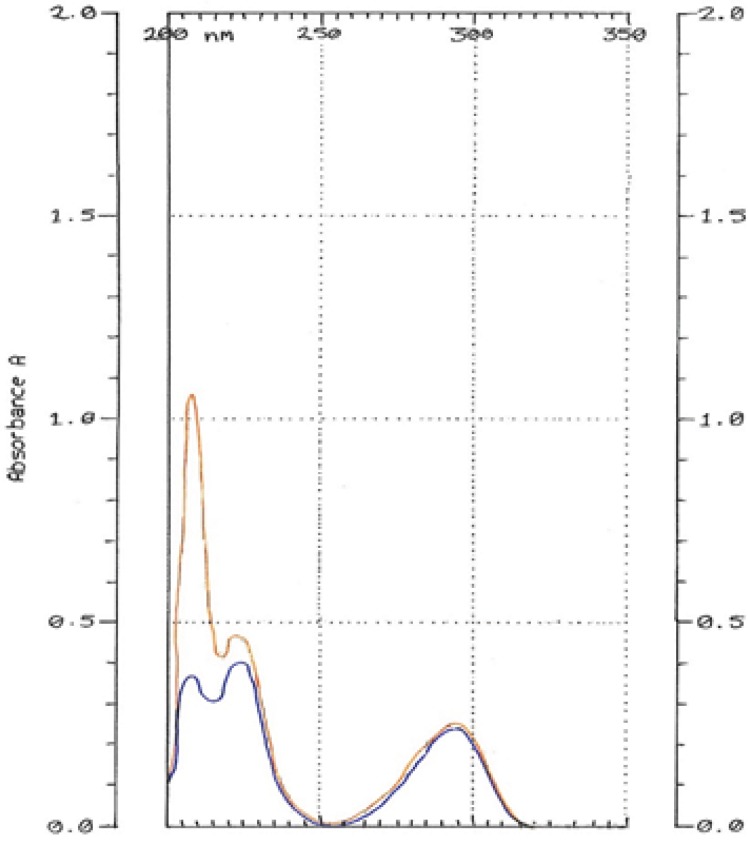
Hydroquinone standard solution (10 µg/mL) mixed with Hydroquinone-free liposome solution diluted 500 times (orange). Hydroquinone standard solution in 10 µg/mL concentration (blue)

HQ-free Liposome Spectrum in different dilutions was outlined in Figure 4. When it was diluted 500 times, there was no absorption in 293 nm. The 2-spectrum overlay of HQ-free liposome solution with 500 times of dilution and HQ standard solution (10 µg/mL) didn’t show any interface in 293 nm (Figure 5). Also, absorption solution of HQ standard (10 µg/mL) mixed with HQ-free liposome solution diluted 500 times was the same HQ standard solution (Figure 6).

*Accuracy*

The recovery (%) for HQ was102 ± 0.8, 99 ± 0.2 and 98 ± 0.4 for 80%, 100% and 120% respectively. The method is accurate for quantitative estimation of HQ in liposome clearly.

*Precision*

Variation in intra-day and between days (inter-day) was analyzed. By analyzing same concentration of HQ (10 μg/mL), the intra-day and inter-day precision was determined. The study showed precision of inter-day and intra-day as 0.9 and 1.5 % respectively. The RSD (%) value of <2% suggests that the precision is acceptable.

*LOD and LOQ*

LOD and LOQ were found to be 0.24 and 0.72 µg/mL respectively.

## Conclusion

It was concluded from the above results and data that spectrum of liposome in methanol didn’t interfere with HQ in 293 nm if liposome solution was diluted 500 times. The concentration of HQ in liposomal HQ solution was 5 mg/mL; therefore, after being diluted (500 times) by methanol, the HQ concentration reached to10 µg/mL. This dilution was the best dilution for sample preparation in the study of HQ with liposomal matrix by UV spectrophotometry. The molar ratio of HQ and the substances contributed in liposome was an important factor in dilution rate and in removing liposome absorption. The results of validation parameters showed that this method is acceptable for HQ determination in liposome. 
